# Genome-Wide Identification and Expression Profiling of the ABF Transcription Factor Family in Wheat (*Triticum aestivum* L.)

**DOI:** 10.3390/ijms25073783

**Published:** 2024-03-28

**Authors:** Fuhui Yang, Xuelian Sun, Gang Wu, Xiaoyan He, Wenxing Liu, Yongmei Wang, Qingyi Sun, Yan Zhao, Dengan Xu, Xuehuan Dai, Wujun Ma, Jianbin Zeng

**Affiliations:** 1 Shandong Provincial Key Laboratory of Dryland Farming Technology, College of Agronomy, Qingdao Agricultural University, Qingdao 266109, China; 2 Academy of Dongying Efficient Agricultural Technology and Industry on Saline and Alkaline Land in Collaboration with Qingdao Agricultural University, Dongying 257347, China

**Keywords:** wheat (*Triticum aestivum* L.), *TaABFs*, genome-wide analysis, expression patterns, regulatory network, abiotic stress

## Abstract

Members of the abscisic acid (ABA)-responsive element (ABRE) binding factor (ABF) and ABA-responsive element binding protein (AREB) families play essential roles in the regulation of ABA signaling pathway activity and shape the ability of plants to adapt to a range of stressful environmental conditions. To date, however, systematic genome-wide analyses focused on the ABF/AREB gene family in wheat are lacking. Here, we identified 35 *ABF/AREB* genes in the wheat genome, designated *TaABF1*–*TaABF35* according to their chromosomal distribution. These genes were further classified, based on their phylogenetic relationships, into three groups (A–C), with the *TaABF* genes in a given group exhibiting similar motifs and similar numbers of introns/exons. Cis-element analyses of the promoter regions upstream of these *TaABFs* revealed large numbers of ABREs, with the other predominant elements that were identified differing across these three groups. Patterns of *TaABF* gene expansion were primarily characterized by allopolyploidization and fragment duplication, with purifying selection having played a significant role in the evolution of this gene family. Further expression profiling indicated that the majority of the *TaABF* genes from groups A and B were highly expressed in various tissues and upregulated following abiotic stress exposure such as drought, low temperature, low nitrogen, etc., while some of the *TaABF* genes in group C were specifically expressed in grain tissues. Regulatory network analyses revealed that four of the group A *TaABF*s (*TaABF2*, *TaABF7*, *TaABF13*, and *TaABF19*) were centrally located in protein–protein interaction networks, with 13 of these *TaABF* genes being regulated by 11 known miRNAs, which play important roles in abiotic stress resistance such as drought and salt stress. The two primary upstream transcription factor types found to regulate *TaABF* gene expression were BBR/BPC and ERF, which have previously been reported to be important in the context of plant abiotic stress responses. Together, these results offer insight into the role that the *ABF/AREB* genes play in the responses of wheat to abiotic stressors, providing a robust foundation for future functional studies of these genes.

## 1. Introduction

Wheat (*Triticum aestivum*) is among the most extensively cultivated crops globally, accounting for 30% of worldwide cereal production [1]. It is a primary source of essential nutrients and energy for humans, serving as the source of ~15% of daily caloric intake [2,3]. While there has been a growing focus on the importance of maintaining food security from both consumer groups and the scientific community in recent years, efforts to effectively improve crop productivity under adverse environmental conditions remain challenging [4]. Wheat is sensitive to an array of abiotic stressors, most notably high soil salinity and drought conditions, which can result in yields that are unpredictable and corresponding reductions in agronomic output [5]. When faced with these adverse conditions, plants engage in a range of physiological, metabolic, and molecular responses that can help them survive and thrive under these unfavorable conditions [6]. Many different transcription factor (TF) families serve as key regulators of gene expression in these contexts through their ability to bind to target gene promoters, thereby serving as vital regulators of plant tolerance to drought and salinity [7].

The basic leucine zipper (bZIP) family of TFs is among the best-conserved and largest plant TF families, playing key roles in a range of developmental processes and in the ability of plants to respond to biotic and abiotic stressors [8]. Plant bZIP TFs contain a highly conserved 60–80 amino acid (aa) bZIP domain comprising a basic DNA binding domain as well as a leucine zipper domain [9,10]. The basic region, which has an N-X7-R/K amino acid sequence, is involved in localization to the nucleus and binding to specific sequences of DNA, activities which exhibit a high degree of conservation in eukaryotes [11,12]. In contrast, there is greater variability with respect to the composition of the leucine zipper domain, which consists of the repeating L-X6-L-X6-L sequence with leucine as every seventh aa that may, in some instances, be substituted by other bulky hydrophobic amino acids including isoleucine, phenylalanine, valine, or methionine [13]. The resultant leucine zipper produces an amphiphilic α helical structure that facilitates bZIP protein dimerization prior to DNA binding [14]. The basic functionality of these bZIP TFs entails their leucine zipper-mediated dimerization. These plant bZIP proteins also harbor other conserved motifs, including proline, glutamine, and acidic amino acid enrichment domains involved in bZIP gene transcriptional activation [11]. In most cases, these bZIP proteins preferentially engage ACGT sequences as a core *cis*-element to form a palindromic structure, particularly for C-boxes (GACGTC), G-boxes (CACGTG), and A-boxes (TACGTA) [15,16].

As the genomic characteristics of plants have been increasingly decoded, a growing number of gene families encoded therein have been identified and characterized. Many bZIP TF family members have been isolated to date across a wide number of plant species, including Arabidopsis [11], alfalfa [17], barley [18], cassava [19], castor bean [20], soybean [21], cucumber [22], grapevine [23], legume [24], maize [25], olive [26], rice [27], and sorghum [28]. These include the abscisic acid (ABA)-responsive element (ABRE) binding factor (ABF) and ABA-responsive element binding protein (AREB) (ABF/AREB) subfamily of bZIP TFs, which are essential regulators of the expression of downstream stress-responsive genes in pathways associated with ABA [29,30]. These ABF/AREB proteins belong to the group A bZIP TF subfamily and modulate stress-adaptation-related gene expression through their ability to bind to ABRE cis-acting elements [31]. Prior studies have characterized several genes in this ABF/AREB subfamily (e.g., *ABF1*, *AREB1/ABF2*, *ABF3*, and *AREB2/ABF4*), highlighting their importance as key TFs involved in ABA signaling pathways in response to abiotic stressors including salinity and drought [32]. Overexpressing *BnaABF2* in Arabidopsis, for example, can improve tolerance to salinity and drought through the regulation of a range of ABA-dependent stress signaling genes including *RD29B*, *RAB18,* and *KIN2* [33]. ABA can reportedly induce the expression of the *StABF1*, as can drought, salinity, and cold stress, suggesting that it may function as a key regulator of ABA-dependent stress signaling pathway activity in potato plants [34]. *PtrABF* overexpression in tobacco offers improved tolerance to drought and dehydration attributable to increased antioxidant enzyme levels and a corresponding decrease in reactive oxygen species accumulation [35]. *GhABF3* expression can reportedly be induced in response to treatment with ABA or exposure to salinity and drought [36]. *GhABF3* overexpression can significantly bolster the ability of cotton and Arabidopsis plants to tolerate salinity and drought [36]. A number of other ABF/AREB subfamily genes have also been shown to improve the ability of transgenic plants to tolerate abiotic stressors, particularly salinity and drought, as in the cases of *ABF2* in *Vitis vinifera* [37], *ABF3* and *ABF4* in Arabidopsis [38,39], *ABF2* in rice [40], and *ABF2D* and *ABF3* in cotton [36,41].

Given that there have been many studies demonstrating that subfamily A bZIP members (ABF/AREB proteins) serve as important mediators of ABA signaling and regulators of abiotic stress responses in plants, they may offer substantial value as targets for efforts aimed at augmenting wheat tolerance for abiotic stress conditions. However, research focused on this wheat gene family has been insufficiently comprehensive to date. Here, a systematic investigation led to the identification and characterization of 35 members of the wheat ABF/AREB TF family, including analyses of conserved motifs, gene structures, chromosomal distributions, phylogenetic relationships, promoter cis-acting regulatory elements, and collinearity within and among species. Transcriptomic profiling was also conducted to examine *TaABF* gene expression across tissues and in response to conditions including heat, drought, cold, salt, N starvation, and P starvation. Interactions among these TaABF proteins and their associated regulatory networks were additionally predicted. Together, these findings offer a valuable foundation for efforts to understand the molecular functions and characteristics of the wheat *TaABF* TF family, offering a reference for future genetic breeding efforts and studies of the biological roles played by these proteins.

## 2. Results

### 2.1. Identification of Members of the Wheat ABF TF Family

To identify all ABF genes in the wheat genome, the Blastp program was initially employed to search the wheat protein database based on nine ABF/AREB sequences of Arabidopsis. After using the SMART, NCBI-CDD, and Pfam databases to confirm the structural domain integrity, a total of 35 *TaABF* genes were identified in the wheat genome, which were numbered according to their chromosomal locations (*TaABF1*–*TaABF35*, Table S1). The coding sequences, molecular weights (MWs), and the isoelectric points (pIs) corresponding to these genes were next analyzed from silico studies (Table S1). All 35 TaABFs were from 223 aa (TaABF24) to 391 aa (TaABF8, TaABF15, TaABF16, and TaABF17), with an average length of 339 aa. These proteins ranged from 24.52 kDa (TaABF24) to 42.40 kDa (TaABF15) in size, with an average of 36.75 kDa. The isoelectric point (pI) values ranged from 4.96 (TaABF17) to 10.49 (TaABF24), with an average of 7.97, and 60% exhibited a pI > 7.

**Figure 1:**
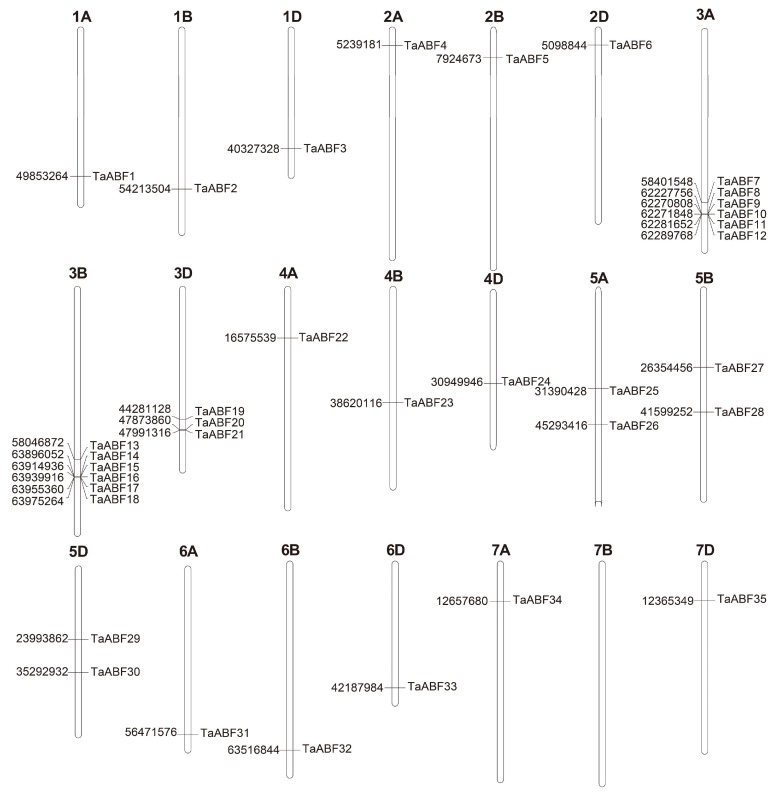
Chromosomal *TaABF* gene locations. The numbers on the left correspond to the physical locations of *TaABF* genes on the chromosomes, and 1A to 7D at the top are the names of each chromosome.

Members of the 35 *TaABF* gene family were distributed across the 20 wheat chromosomes with the exception of Chr7B. The largest number of these *TaABFs* were distributed on Chr3 (*n* = 15, 42.8%), 6 on Chr3A, 6 on Chr3B, 3 on Chr3D, and 6 on Chr6 (17.1%), while few were present on Chr7 (2, 5.7%), and 3 each were found on Chr1, Chr2, Chr4, and Chr6 (Figure 1).

### 2.2. Phylogenetic Analyses and Classification of Wheat ABF TF

**Figure 2:**
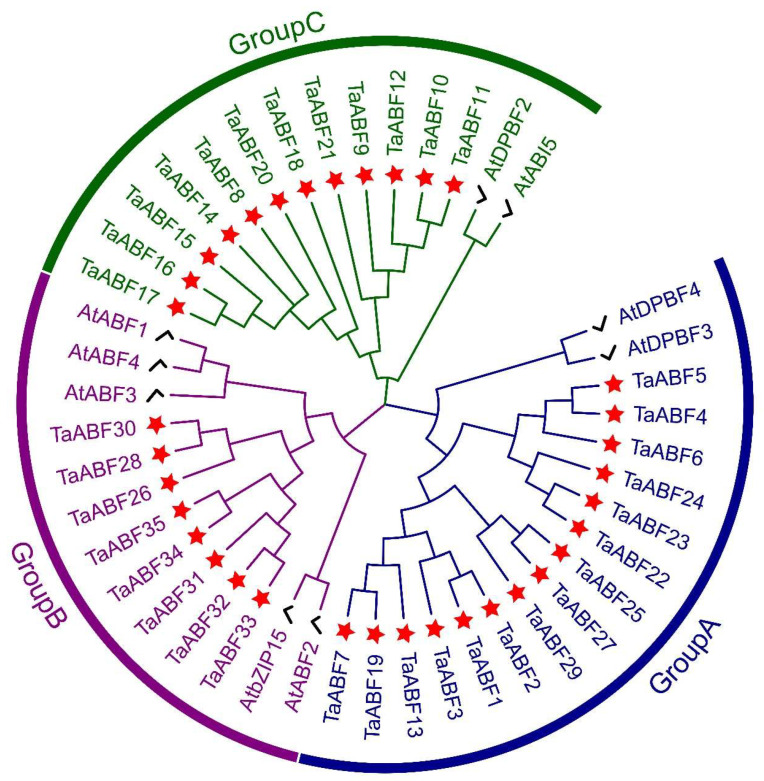
Phylogenetic tree corresponding to ABF gene families from Arabidopsis and wheat. MEGA-11 was used for multiple sequence alignment, after which the Maximum Likelihood (ML) method with 1000 replicates was employed for tree construction. Blue, purple, and green lines, respectively, correspond to the branches harboring group A, B, and C TaABF family members. Red stars and black checkmarks are, respectively, used to denote TaABFs and AtABFs.

To explore the evolutionary relationships among the ABF family members from wheat and Arabidopsis and to inform their classification, the ML method was used to construct an unrooted phylogenetic tree based on the full-length protein sequences corresponding to these 35 *TaABF*s genes (Table S2) and 9 *AtABF* genes (Table S3). According to support values (85% or greater) of the phylogenetic tree, these 35 *TaABF*s and 9 *AtABFs* were separated into three subgroups (Figure 2), with all three groups including both *AtABFs* and *TaABFs*. Group A was the largest of these subfamilies, including 15 *TaABF*s, followed by Group C with 12 members and Group B with 8 members. In Arabidopsis, the nine ABF/AREB family members were phylogenetically divided into two subfamilies, the ABF/AREB subfamily (AtABF1, AtABF2/AREB1, AtABF3, and AtABF4/AREB2) and the AtDPBF/ABI5 subfamily (AtDPBF1/ABI5, AtDPBF2, AtAREB3/DPBF3, AtDPBF4/EEL, and AtbZIP15). The *TaABFs* in Group B had high similarity with the ABF/AREB subfamily members, while the *TaABFs* in Group A and C showed high similarity with the AtDPBF/ABI5 subfamily members.

### 2.3. Conserved Motif and Gene Structure Analyses of Wheat TF

**Figure 3:**
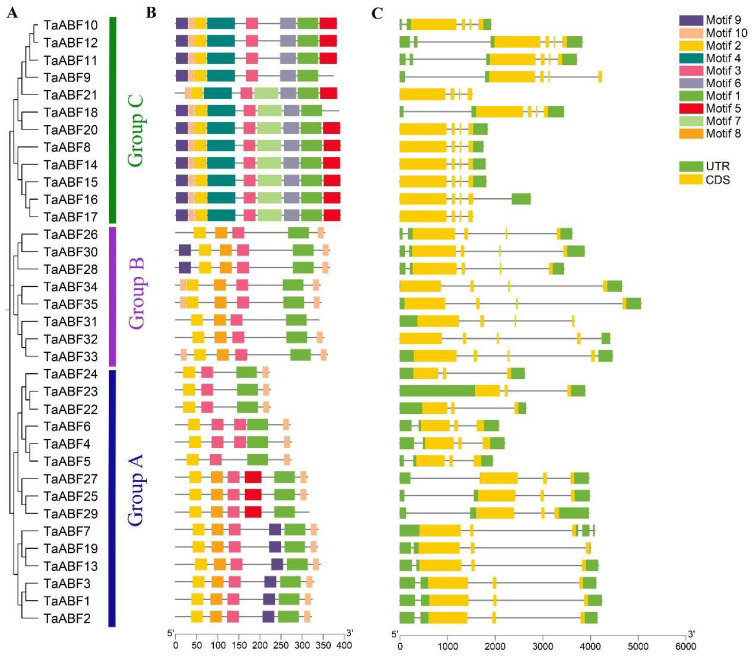
Analyses of *TaABF* gene structures and motifs. (**A**) A phylogenetic tree corresponding to the evolution of the wheat *TaABF* gene family. (**B**) Conserved motifs within *TaABF* genes. (**C**) *TaABF* exon/intron gene structures.

Further analyses of the *TaABF* gene sequence characteristics were next conducted by assessing the conserved motif distributions and exon–intron structure characteristics. The MEME program led to the identification of 10 total conserved motifs among these TaABFs, which were subsequently annotated using InterPro (Table S4). A motif distribution corresponding to the established phylogenetic tree (Figure 3A) for members of the TaABF family is presented in Figure 3B. Motif numbers ranged between 4 and 8, with Motif 1 corresponding to the conserved bZIP domain that was present in all the annotated TaABFs, whereas Motifs 4, 6, 7, and 8 correspond to variable motifs, of which Motifs 4, 6, and 7 were only apparent in the Group C TaABFs while Motif 8 was only found in Groups A and B. Motif 9 was present in all Group C family members other than TaABF21, whereas just six and two members of Groups A and B, respectively, harbored this motif. Motif 5 was present in most Group C members other than TaABF9 and TaABF18, while this motif was only found in three members of Group A. Gene structure analyses revealed introns in all the *TaABF* genes, with a range of 2–5 introns per gene (Figure 3C). Similar lengths and exon/intron numbers were observed for members of the same Group, with members of Group A generally exhibiting 2–4 introns while Group B and C members exhibited 3–5 introns.

### 2.4. Expansion Pattern Analyses of TaABF Gene

**Figure 4:**
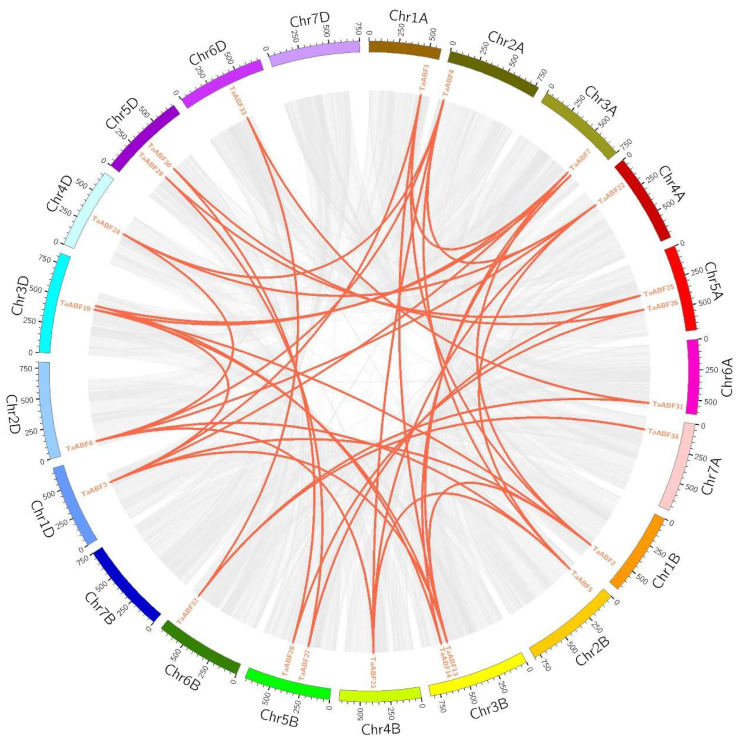
Wheat TaABF family synteny analyses. Syntenic gene pairs in the wheat genome are denoted using gray lines, while syntenic *TaABF* gene pairs are denoted with red lines. Chromosome numbers are indicated, and the lengths of chromosomes are indicated with the corresponding scales.

**Figure 5:**
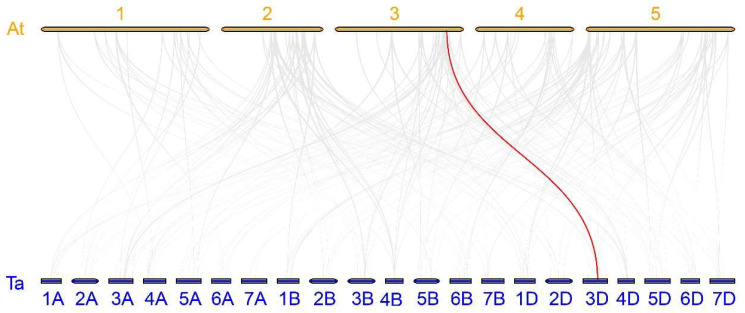
*TaABF* gene synteny analyses in Arabidopsis (At) and wheat (Ta). Homologous gene pairs in the wheat and Arabidopsis genomes are marked with gray lines, while the *TaABF* gene pair exhibiting homology in the wheat and Arabidopsis genomes is marked with red line. Numbers 1 to 5 are the chromosome names of Arabidopsis. 1A to 7D are the chromosome names of wheat.

To evaluate the mechanisms through which the wheat *TaABF* gene family has expanded with type, syntenic analyses were conducted for these 35 *TaABF*s by using McscanX with a blast e-value = 1 × 10^−5^. In total, synteny was observed for 40 *TaABF* gene pairs, all of which were located on different chromosomes (Figure 4, Table S5). These included 13, 11, and 10 gene pairs observed between the A and B, A and D, and B and D subgenomes, respectively. Two collinear gene pairs were detected within subgenomes A, B, and D. These results suggest that segmental duplication is the primary driver of wheat TaABF gene family expansion, with duplication events primarily taking place among the A, B, and D subgenomes without occurring within the same subgenome. In addition, three *TaABF* genes (*TaABF1*, *TaABF2*, and *TaABF4*) exhibited more than four syntenic pairs. *TaABF4* on Chr2A, for instance, had synteny with *TaABF5* (Chr2B), *TaABF6* (Chr2D), *TaABF22* (Chr4A), *TaABF23* (Chr4B), and *TaABF24* (Chr4D), suggesting an important role for this gene in the context of wheat ABF gene family expansion. A synteny analysis was also conducted between the Arabidopsis and wheat genomes, revealing just one homologous gene pair (*TaABF19*/*AtDPBF3*) (Figure 5, Table S6).

As a further approach to assessing the evolutionary constraints acting on this gene family, the Ks and Ka values and the Ka:Ks ratio were assessed, as was the divergence time for each ABF gene pair. Ka/Ks < 1 represents a negatively selected gene, Ka/Ks > 1 indicates a positively selected gene, and Ka/Ks = 1 represents a neutrally selected gene. The Ka:Ks ratio values for most of the segmental duplicated *TaABF* gene pairs were <1, other than the *TaABF29*/*TaABF27* pair with a value of 1.02, consistent with a more important role for purifying selection in the context of TaABF gene family evolution, with the divergence time occurring between 2.53 Mya and 53.74 Mya (Table S7).

### 2.5. Analyses of Cis-Acting Regulatory Element Analyses in TaABF Genes

**Figure 6:**
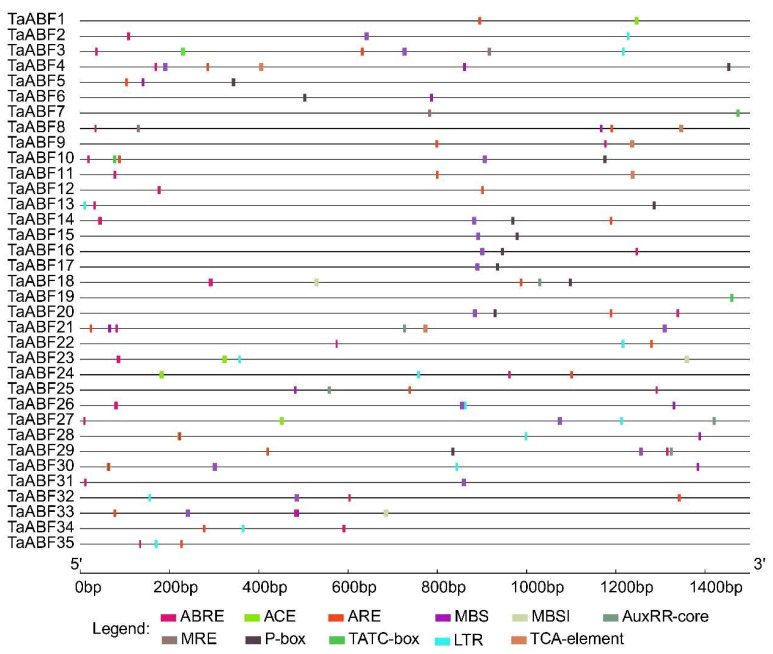
Analyses of cis-acting elements in wheat *TaABF* gene promoters by using PlantCARE. The scale along the bottom denotes the relative position upstream of the translation start site for identified elements.

The potential functional roles of *TaABF* genes were next explored by conducting a cis-acting element analysis of the promoter regions upstream of these 35 *TaABF*s, scanning for elements such as ABREs, light-response-related ACE elements, drought-inducible MBS elements, auxin-responsive AuxRR-core elements, anaerobic-inducible ARE elements, low-temperature-responsive LTR elements, gibberellin-responsive P-box elements, and salicylic-acid-responsive TCA elements (Figure 6, Table S8). These analyses led to the identification of ABRE-, ARE-, and TCA-element sequences across all three groups, with the promoters of 28 *TaABF*s (80%) harboring ABRE elements, 21 (60%) harboring ARE elements, and 17 *TaABF*s (49%) harboring TCA elements. In contrast, AuxRR-core and P-box elements were only detectable in Group A and C members while ACE and LTR elements were only present in Group A and B members, and MBS elements were primarily restricted to members of Groups A and B.

### 2.6. Analyses of TaABF Gene Expression in Response to Spatial and Abiotic Stressors

**Figure 7:**
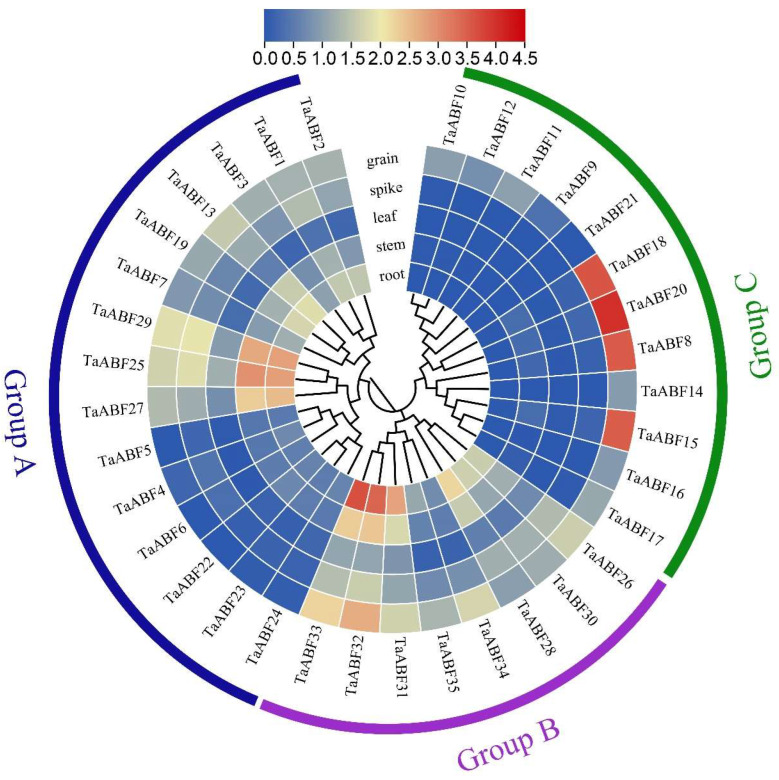
Characterization of tissue-specific patterns of *TaABF* gene expression using the heatmap tool of Tbtools v1.09. Results were analyzed using the log2(TPM + 1) format, with redder and bluer coloration, respectively, corresponding to higher and lower levels of expression.

To further explore ABF gene expression across wheat tissue types, expression data for 35 *TaABF* were downloaded for the root, leaf, grain, spike, and stem tissues from the China Spring wheat variety using the WheatOmics 1.0 database. Significant variations in *TaABF* gene expression patterns were noted in the resultant heat map (Figure 7, Table S9). All members of Group B and half of the Group A family members were highly expressed in various tissues, while most Group C *TaABFs* were only expressed at low levels across all tissues with the exception of *TaABF8*, *TaABF15*, *TaABF18*, and *TaABF20*, which exhibited high expression levels in wheat grains.

**Figure 8:**
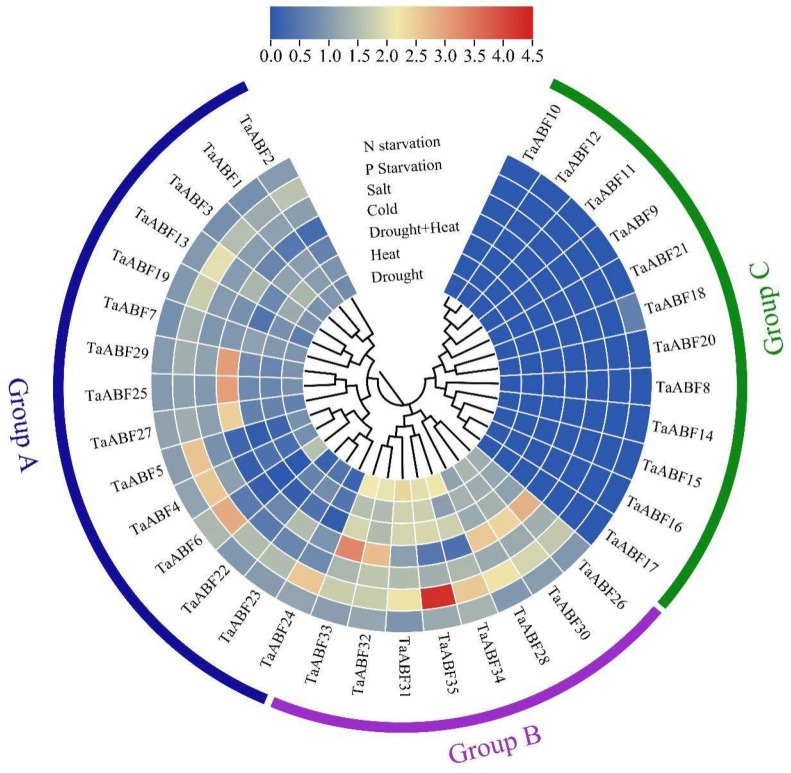
Analyses of *TaABF* gene expression in response to various abiotic stressors using the heatmap tool of Tbtools v1.09. Data were analyzed using the log2(fold change + 1) format, with redder and bluer, respectively, corresponding to higher and lower levels of expression.

Using published wheat transcriptomic data, analyses of *TaABF* gene responses to a range of abiotic stressors including salinity, cold, heat, drought, and N or P starvation were assessed. These analyses revealed significant variability with respect to *TaABF* gene expression under these different stress conditions and across the three subgroups of these genes (Figure 8, Table S10). In general, the Group C *TaABF* genes were downregulated or unchanged in response to these abiotic stressors, whereas the Group A and B *TaABFs* tended to be upregulated by stress exposure, with greater upregulation for Group B genes in most cases. *TaABF33*, for example, exhibited, respectively, 2.15-, 1.52-, 1.86-, 3.29-, 1.40-, 1.73-, and 1.03-fold upregulation in response to drought, heat, drought + heat, cold, salt, P starvation, and N starvation. Strikingly, some Group A and B *TaABF* genes exhibited pronounced upregulation under conditions of P starvation or cold stress exposure, as in the cases of *TaABF35*, which was upregulated by 4.31-fold by P starvation, and *TaABF29*, which was upregulated by 3.03-fold by cold stress exposure.

**Figure 9:**
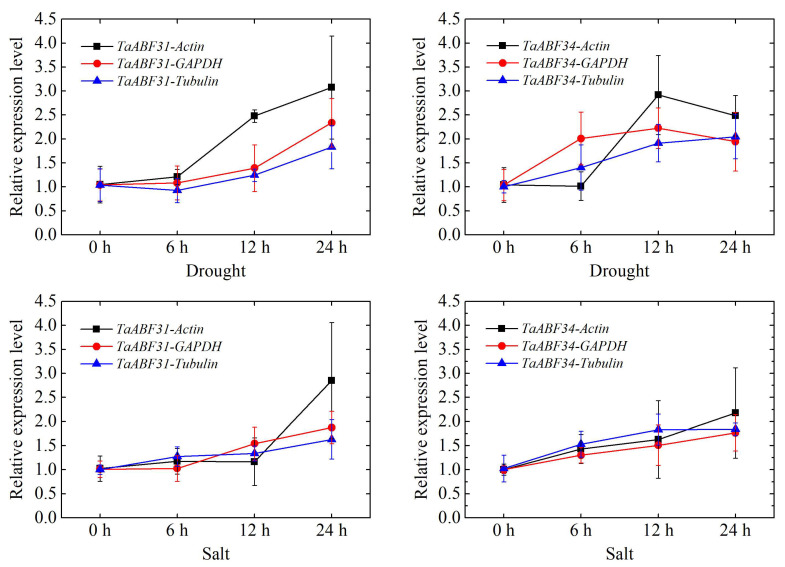
*TaABF31* and *TaABF34* expression patterns under conditions of salt and drought stress. *TaABF31-Actin*, *TaABF31-GAPDH,* and *TaABF31-Tubulin* represent *TaABF31* expressions by using *Actin*, *GAPDH*, and *Tubulin* as internal references, respectively. *TaABF34-Actin*, *TaABF34-GAPDH,* and *TaABF34-Tubulin* represent *TaABF34* expressions by using *Actin*, *GAPDH*, and *Tubulin* as internal references, respectively. Treatment duration is marked along the *x*-axis, while expression levels are marked along the *y*-axis. Expression data are presented as the mean data from three biological replicates.

To verify *TaABF* gene expression patterns, the group B *TaABF31* and *TaABF34* genes, which were upregulated in response to salt and drought stress exposure, were chosen for qPCR-based validation (Figure 9) using primers from Table S11. These analyses confirmed that significant *TaABF31* and *TaABF34* upregulation were evident when exposed to drought stress, with respective fold upregulation values of 2.48 and 3.07, 2.92 and 2.49 at 12 and 24 h of drought stress by using *Actin* as an internal reference. At 24 h of salt stress exposure, these two genes were also upregulated by 2.85- and 2.18-fold, respectively, using *Actin* as an internal reference. When using *GAPDH* and *Tubulin* as internal reference genes, the same expression trends of *TaABF31* and *TaABF34* were also detected.

### 2.7. TaABF Gene Regulatory Networks

**Figure 10:**
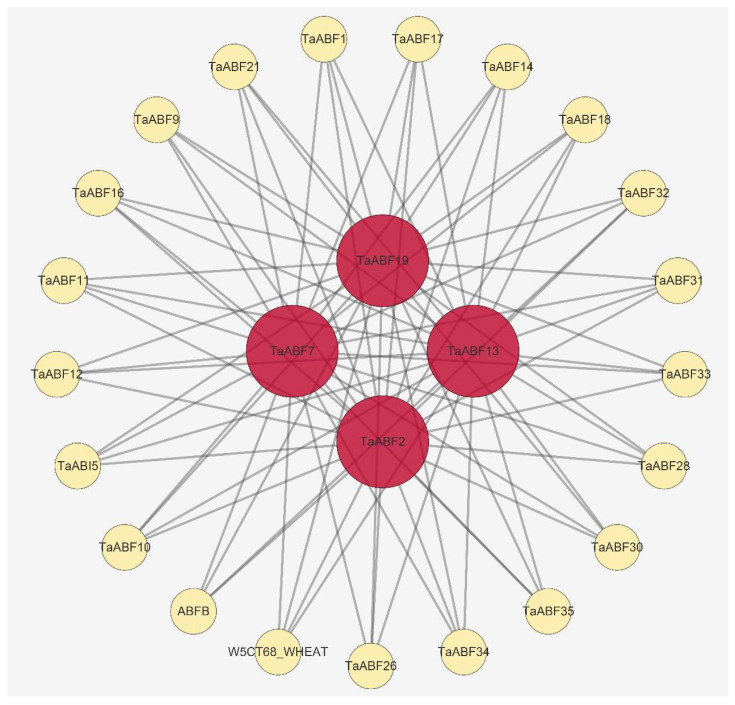
Predictive PPI network model corresponding to members of the TaABF protein family through the use of STRING database. Target circles with deeper red coloration are indicative of more interactions with other protein targets.

The STRING database was next leveraged to predict protein–protein interactions among the TaABF family members. In total, 84 interaction pairs with combined scores >0.5 were identified (Figure 10, Table S12). Of the interacting proteins identified in these analyses, four of the centrally located proteins were encoded by Group A genes (*TaABF2*, *TaABF7*, *TaABF13*, and *TaABF19*). All Group B members and nine (75%) Group C members were predicted to interact with the proteins encoded by these four *TaABF* genes, suggesting that they exert specific regulatory functions through their ability to interact with other *TaABFs*.

**Figure 11:**
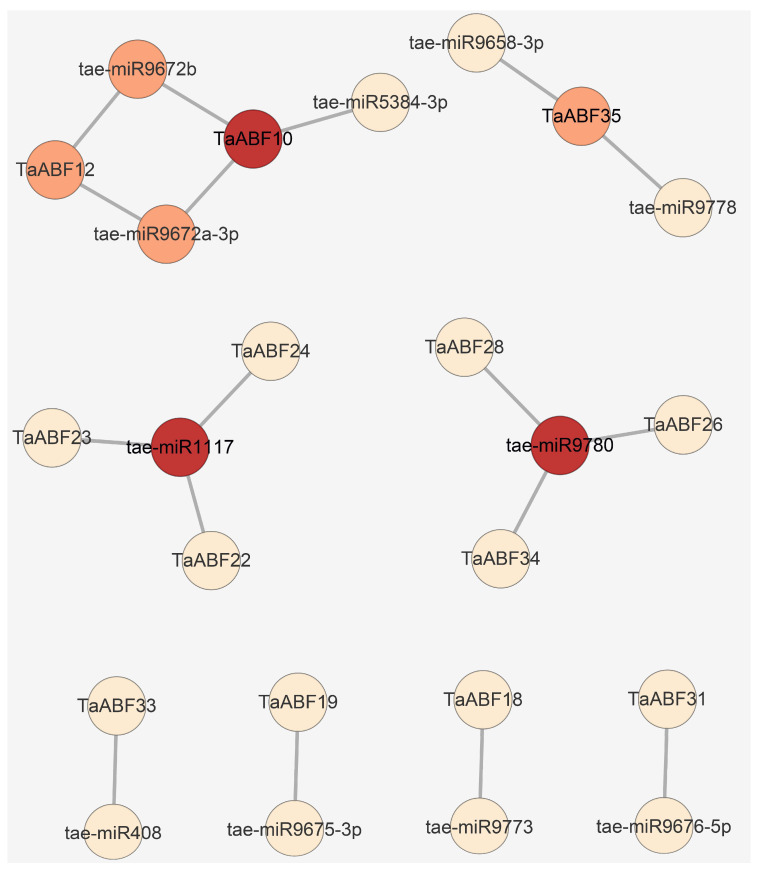
Prediction of interaction networks between miRNAs and *TaABFs* via psRNATarget server. Redder coloration is indicative of a higher number of interactions between miRNAs and target *TaABFs*.

To probe the potential wheat miRNA-*TaABF* regulatory networks, a scan of 119 known wheat miRNAs was conducted, ultimately leading to the identification of 11 that were predicted to serve as inhibitors of *TaABF* transcription (Figure 11, Table S13). In general, the *TaABFs* targeted by these miRNAs were separated into three types, including Type I targets for which one miRNA served as a regulator for one *TaABF* (e.g., tae-miR408:TaABF33, tae-miR9675-3p:TaABF19, tae-miR9773:TaABF18, and tae-miR9676-5p:TaABF31), Type II targets for which one miRNA served as a regulator for three *TaABFs* (e.g., tae-miR1117:TaABF22/TaABF23/TaABF24 and tae-miR9780:TaABF26/TaABF28/TaABF34), and Type III targets for which multiple miRNAs regulated a single *TaABF* (e.g., tae-miR9672b/tae-miR9672a-3p:TaABF12, tae-miR9672b/tae-miR9672a-3p/tae-miR5384-3p:TaABF10, and tae-miR9658-3p/tae-miR9778:TaABF35).

**Figure 12:**
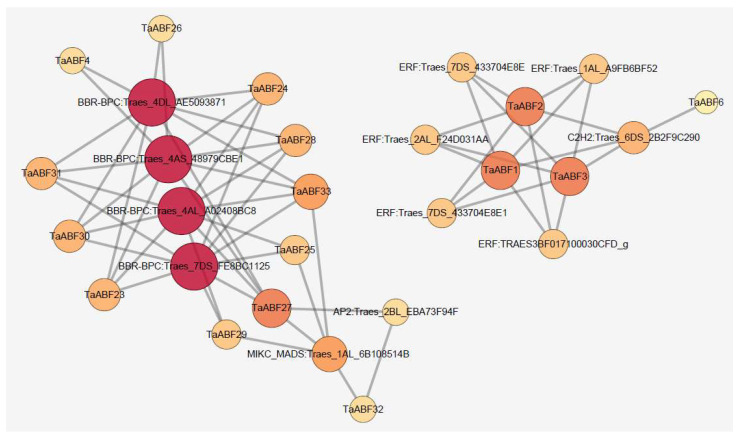
Predictive analyses of the upstream TFs associated with *TaABF* genes by using PlantRegMap tool. Larger and redder circles denote more regulatory relationships with the indicated genes.

To further clarify the regulatory TFs that govern *TaABF* expression in wheat, predictive analyses of upstream TFs were conducted. In total, 63 binding sites for five TFs (BBR-BPC, ERF, MIKC-MADS, AP2, and C2H2) were detected within the promoters upstream of 16 *TaABF*s, with BBR-BPC and ERF binding sites being the most common, regulating the expression of 11 and 3 *TaABF*s, respectively (Figure 12, Table S14). All of the *TaABF* genes for which regulatory TFs were identified were members of either Group A (*n* = 10) or Group B (*n* = 6).

## 3. Discussion

ABF/AREB family members are key TFs that regulate ABA-dependent genes and shape abiotic stress and hormone responses in plants [39,42]. No prior studies, however, have entailed detailed analyses of the wheat ABF/AREB gene family. Here, 35 *ABF* genes were identified within the wheat genome, with their sequences being used to separate them into three groups (Figure 2 and Figure 3). Primary drivers of the expansion of this gene family have been found to include genome polyploidization, segmental duplication, tandem duplication, reverse transcription transposition, or exon duplication and reorganization [43,44]. Efforts to understand the evolutionary relationships among members of this gene family are vital to fully unraveling the functions of these genes. Accordingly, chromosomal localization, collinearity, and Ka/Ks analyses of this *TaABF* gene family were conducted. While uneven distributions of these 35 *TaABF*s were observed across 21 chromosomes, they exhibited a more even distribution across the A (13), B (12), and D (10) subgenomes (Figure 1). This suggests a role for chromosomal polyploidization in *TaABF* family expansion. *TaABF* gene synteny was observed among chromosomes, primarily among different subgenomes, whereas it was rarely evident within a given subgenome. Indeed, 85% of the syntenic gene pairs were evident among the A–B, A–D, and B–D subgenomes (Figure 4, Table S5). This suggests that wheat *TaABF* family expansion is primarily the result of segmental duplication. The divergence time ranged from 2.53 to 53.74 Mya, with most Ka/Ks values being < 1 (Table S7), suggesting that during the evolutionary process, members of this *TaABF* gene family have been subjected to robust purifying selective pressure.

Cis-acting elements within promoter regions are vital for the control of gene expression, particularly in the context of abiotic and biotic stress [45]. Analyses of the cis-acting elements found within *ABF* promoter regions thus offer potential opportunities to reveal the associated regulatory network. Here, various cis-acting elements responsive to abiotic stress and hormones were identified in the promoter regions upstream of these *TaABF* genes (Figure 6, Table S8), suggesting that *TaABFs* may be vital for wheat adaptation to abiotic stress and hormonal stimulation. The ABF/AREB family has been shown to be sensitive to ABA responses [46,47]. Overexpressing *TaAREB3* in Arabidopsis improves osmotic and cold tolerance and increases sensitivity to ABA [48]. Of the *TaABF* genes identified herein, 80% were found to exhibit promoters harboring ABRE elements, supporting an important role for *TaABF* genes in the ABA signaling pathway. Significant variations in element types and distribution characteristics were observed among the *TaABFs* in Groups A, B, and C, with auxin- and gibberellin-responsive elements (AuxRR-core and P-box) only being present in Groups A and C, whereas drought- and cold-responsive elements (MBS and LTR) were only present in Groups A and B (Figure 6, Table S8), in line with the transcriptomic results for this gene family (Figure 7 and Figure 8, Tables S9 and S10), suggesting that the *TaABFs* in these different subgroups may exert a range of functions.

MicroRNAs (miRNAs) are small RNAs capable of repressing the expression of genes through binding to highlight complementary target mRNA sequences [49]. A large number of these miRNAs are thought to be evolutionarily ancient, functioning as key regulators of programs of gene expression vital to the control of plant development, survival, and responses to abiotic or biotic stressors [50,51,52,53]. For example, tae-miR1117 has been established as a potent regulator of heat stress and drought responses [54,55], while tae-miR5384-3p is upregulated in mycorrhized wheat leaves and functions by targeting the gene encoding nucleoredoxin 1, which is an antioxidant enzyme that plays protective roles in the context of oxidative stress [56,57]. There is also evidence supporting the important role that tae-miR9778 plays in key metabolic pathways in young wheat spikes and tiller primordia [58]. Here, several *TaABFs* were predicted to be regulatory targets of these and other miRNAs (Figure 11, Table S13), supporting prominent links between these TF-encoding genes and miRNA-mediated regulation.

There is growing evidence supporting the potential inefficiency of ABF/AREB proteins alone as transcriptional regulators of ABA-responsive genes, with physical interactions between these ABF/AREB family members and other TFs being essential for the synergistic regulation of osmotic stress and dehydration-related plant responses [59,60]. In Arabidopsis, for instance, the TF IDD14 is capable of interacting with *ABF1–4* and cooperatively regulating ABA-mediated tolerance for drought conditions [61]. The plant-specific Barley B Recombinant/Basic PentaCysteine (BBR/BPC) family of GAGA-motif-binding TFs exhibits a high degree of evolutionary conservation across lower and higher plants [62,63,64]. While first identified as playing a role in the context of the development of the endosperm, seed, and ovule [65], these BBR/BPCs were later found to play diverse roles throughout the plant life cycle [66]. *bpc4bpc6* double mutant Arabidopsis plants, for example, present with an array of phenotypes including shorter plant height, fewer rosette leaves, smaller leaves, floral organ defects, and early flowering as compared to wild-type Arabidopsis [67]. BPC1/BPC2 is capable of binding the *GALACTAN SYNTHASE 1* (*GALS1*) promoter and regulating salinity tolerance in Arabidopsis [68]. BPC2 can directly interact with and repress *LATE EMBRYOGENSIS ABUNDANT4-5* (*LEA4-5*) promoter activation, thereby serving as a negative regulator of osmotic stress [69]. Here, six Group A and five Group B *TaABF* genes were found to bind to BBR/BPC TFs (Figure 12, Table S14), suggesting potentially important roles for *TaABFs* in the context of BBR/BPC TF regulation. The Ethylene Responsive Factor (ERF) subfamily is the largest subset of plant APETALA 2 (AP2)/ERF superfamily members, and the biological roles that these proteins play have been widely studied [70]. Subfamily members have been demonstrated to be capable of binding to target gene promoter regions, thereby governing the ability of plants to resist abiotic stressors including drought [71], heat [72], salinity [73], cold [74], and certain other conditions [75,76]. Here, ERF was identified as a key regulatory TF upstream of many of these *TaABFs* (Figure 12, Table S14), providing support for the specific functionality of these *TaABF* genes in the context of resistance to abiotic stress at least in part through ERF-mediated regulatory activity.

## 4. Materials and Methods

### 4.1. Wheat TaABF Family Member Identification

Conserved amino acid sequences for nine ABF/AREB family members from *Arabidopsis thaliana* (AtABF1, AtABF2/AREB1, AtABF3, AtABF4/AREB2, AtDPBF1/ABI5, AtDPBF2, AtAREB3/DPBF3, AtDPBF4/EEL, and AtbZIP15) were downloaded from TAIR (https://www.arabidopsis.org/, accessed on 21 August 2023). Wheat (*Triticum aestivum* L.) genomic sequences and corresponding annotation files were obtained from the Ensembl Plants database (http://plants.ensembl.org, accessed on 25 August 2023). Sequences corresponding to Arabidopsis ABF/AREB protein sequences were identified using the Blastp program to search the wheat genome, using Arabidopsis sequences as reference queries. To reduce false positive results, an E-value of 1 × 10^−20^ was utilized. To facilitate additional screening, candidate protein sequences were submitted to the SMART (http://smart.embl.de/, accessed on 5 September 2023), NCBI-CDD (https://www.ncbi.nlm.nih.gov/cdd/, accessed on 6 September 2023), and Pfam (http://pfam.xfam.org/, accessed on 7 September 2023) databases, removing any sequences that were incomplete or failed to match the core structural domain (PF00170) sequences, thereby yielding the final *TaABF* family members. ExPASy (http://web.expasy.org/protparam/, accessed on 10 September 2023) was then employed to calculate the amino acid length, molecular weight (MW), and isoelectric point (pI) for each of the identified *TaABFs*.

### 4.2. Phylogenetic Tree, Gene Structure, Conserved Motif, and Cis-Acting Element Analyses

ClustalW was used for phylogenetic analyses of 35 TaABF proteins and the Arabidopsis ABF proteins, while MEGA11.0 was used to construct a Maximum Likelihood (ML) phylogenetic tree with 1000 replicates. The evolutionary tree was edited with the iTOL v2.0 website (https://itol.embl.de/, accessed on 14 September 2023).

The wheat genome GFF3 file was used to analyze *TaABF* gene exon and intron numbers and gene lengths as assessed with the GSDS tool (http://gsds.cbi.pku.edu.cn/, accessed on 17 September 2023). The MEME tool (http://meme-suite.org/, accessed on 20 September 2023) was employed to analyze conserved motifs within TaABF proteins, with a number of motifs set to 10 and a motif width ranging from 15 to 100. The *TaABF* gene structures and conserved motifs were visualized with Tbtools v1.09 [77].

TBtools v1.09 was used to extract the 2000 bp sequences upstream of the ATG codon for each gene, and these sequences were then submitted to the PlantCARE website (http://bioinformatics.psb.ugent.be/webtools/plantcare/html/, accessed on 23 September 2023) to analyze cis-acting regulatory elements within these promoter regions. The results were then imported into TBtools v1.09 for visualization.

### 4.3. Chromosomal Localization and Gene Duplication Analyses

Wheat genome GFF3 files were used to obtain the positions of the *TaABF* genes on the wheat chromosomes, with the results being visualized with MapChart tools v2.3.2 and genes being named based on their relative positions. The chromosome length, gene density, gene locations, and synteny-related information for these wheat *TaABF* genes were obtained from the wheat genome database to conduct synteny analyses. MCScanX was used to analyze the synteny between the wheat and Arabidopsis ABF genes. The ParaAT tool was employed to calculate the nonsynonymous substitution rate (Ka) and synonymous substitution rate (Ks) values for the *TaABFs* [78], after which Calculator v2.0 was used to compute the Ka:Ks ratio to evaluate the selection pressure, with the model set to the MA model [79]. The divergence time of the collinear gene pairs was estimated as Ks/(2 × 9.1 × 10^−9^), with 9.1 × 10^−9^ as the mutation rate per base per year [80].

### 4.4. TaABF Gene Expression Profiling

The Wheat Omics 1.0 database (http://wheatomics.sdau.edu.cn/, accessed on 2 October 2023) was accessed to download expression data for the wheat leaf, stem, root, grain, and spike tissues or from tissues of wheat plants subjected to different stressors (heat, cold, salt, drought, N starvation, and P starvation) in the transcript per kilobase per million mapped reads (TPM) format, after which expression heatmaps were constructed using log2(TPM + 1) values of 35 *TaABF* genes and plotted with TBtools v1.09.

### 4.5. Verification of TaABF Gene Responses to Salt and Drought Stress

Seeds from the Chinese Spring wheat variety were disinfected for 5 min using 75% alcohol, rinsed 2–3 times using distilled water, and germinated by placing them into Petri dishes containing two layers of moistened test paper for 7 days. These seedlings were then transferred into 5 L black plastic buckets filled with 1/5 Hoagland nutrient solution (pH 5.8–6.0) and incubated with continuous aeration under controlled conditions (16 h/8 h; 23 °C/18 °C; day/night), changing this nutrient solution every 2–3 days. When seedlings exhibited two leaves, drought and salt stress conditions were, respectively, simulated through treatment with 15% (*w*/*v*) PEG 6000 and 200 mM NaCl. A total of three biological replicates were established for all conditions. The roots were either harvested at baseline (0 h) or at 6, 12, or 24 h post-stress initiation. After collection, these samples were snap-frozen with liquid nitrogen and stored at −80 °C prior to analyses of the gene expression.

The root samples were processed to extract the total RNA with the SteadyPure Plant RNA Extraction Kit (Accurate Biology Co., Qingdao, China), after which the Evo M-MLV Reverse Transcription Kit for qPCR (Accurate Biology Co.) was used to prepare cDNA; additionally, the SYBR Green Pro Taq HS Premixed type Kit (Accurate Biology Co.) was employed for qPCR analyses conducted with QuantStudio™ 3 Real-Time PCR Instruments (Applied Biosystems, Thermo Fisher Scientific, Waltham, MA, USA) with the following settings: 95 °C for 30 s and 40 cycles of 95 °C for 5 s and 60 °C for 30 s. A melt curve analysis was conducted from 60 to 95 °C with 0.5 °C increments (5 s per increment). Actin, GAPDH, and Tubulin were selected as internal references for the gene expression analyses, with the 2^−ΔΔCt^ method being employed to measure the relative expression.

### 4.6. Protein–Protein Interaction (PPI), miRNA-TaABFs Interaction, and TF Regulatory Network Predictive Analyses

The protein sequences of TaABFs were uploaded to the STRING database (https://cn.string-db.org/, accessed on 20 October 2023), restricting the target species to wheat. Interactions among these TaABFs were then predictively analyzed, with Cytoscape v3.7.2 being employed for PPI network visualization.

The psRNATarget server (https://www.zhaolab.org/psRNATarget/analysis?function=2, accessed on 25 October 2023) was employed to identify miRNAs predicted to be capable of regulating *TaABF* gene expression using the following settings: a G:U pair penalty of 0.5, a maximum of 2 mismatches in the seed region, and an opening gap penalty of 2. Regulatory networks were then visualized with Cytoscape v 3.7.2.

To gain further insight into the mechanisms governing *TaABF* regulation, upstream TFs predicted to regulate these genes were identified using the online PlantRegMap tool (http://plantregmap.gao-lab.org/binding_site_prediction.php, accessed on 28 October 2023), setting the species to *Triticum aestivum*, with a *p*-value threshold less than 1 × 10^−6^ and a regulatory relationship score greater than 20. Cytoscape v 3.7.2 was subsequently used for TF-*TaABF* regulatory network visualization.

## 5. Conclusions

In conclusion, this study comprised a comprehensive study of the 35 members of the wheat ABF/AREB family (*TaABF1*–*TaABF35*). Based on their amino acid sequences, these 35 TaABFs were categorized into three groups. These *TaABF* genes had undergone several segmental duplication events, which played a dominant role in the expansion of this gene family. These genes were also subjected to a transcriptomic analysis, cis-acting element, and regulatory network analyses. Together, these results offer a sound foundation for future efforts to characterize wheat *ABF*/*AREB* genes, allowing for in-depth studies of their specific functions.

## Data Availability

Data are contained within the article and Supplementary Materials.

## Supplementary Materials

The supporting information can be downloaded at https://www.mdpi.com/article/10.3390/ijms25073783/s1.
